# Relative validity and reproducibility of a semi-quantitative food frequency questionnaire to assess fruit and vegetable consumption in school-aged children

**DOI:** 10.3389/fnut.2022.934295

**Published:** 2022-08-17

**Authors:** Ana Ilić, Ivana Rumbak, Ružica Brečić, Irena Colić Barić, Martina Bituh

**Affiliations:** ^1^Department of Food Quality Control, Faculty of Food Technology and Biotechnology, University of Zagreb, Zagreb, Croatia; ^2^Department of Marketing, Faculty of Economics and Business, University of Zagreb, Zagreb, Croatia

**Keywords:** childhood, food frequency questionnaire, fruit, reproducibility, validity, vegetables, nutrition methodology

## Abstract

**Background:**

Since the beneficial effects of fruit and vegetable (FV) consumption on health are well known due to the synergy of their nutrients and non-nutrients, it is crucial to have good tools to assess the FV intake. A food frequency questionnaire (FFQ) is an adequate method to estimate FV consumption, but it is necessary to relate this dietary method to the geographic and cultural environment. Therefore, this study presented the development of a semi-quantitative FFQ to estimate the FV intake in school-aged children who usually consume cooked homemade and school meals. It also aimed to evaluate the relative validity and reproducibility of the FFQ.

**Methods:**

School-aged children (baseline age 8 years) from 14 primary schools in the city of Zagreb participated in the study during the 2019/2020 school year. Parents/caregivers, together with the children, completed the FFQs and 3-day dietary records (3DDRs). The FFQ was designed to assess the consumption of eight food categories. The FFQ was validated using the 3DDR of 141 children (51.4% of boys), whereas the reproducibility test included the FFQ of 161 children (53.4% of boys).

**Results:**

Of the eight food categories, FFQ overestimates the consumption of three and underestimates the consumption of three food categories (*p* < 0.05; Wilcoxon signed rank test) compared to the 3DDR. De-attenuated correlation coefficients estimated a significant relationship (0.217–0.384) between the FFQ and 3DDR. Cross-classification analysis revealed that overall, 28–41% of children were classified in the same quartile, whereas less than 10% of children were extremely misclassified for all food categories obtained from 3DDR and FFQ1. κ_*w*_ values showed fair agreement for all food categories. The Bland–Altman analysis results showed a relatively small bias for all food categories (median between -11.7 and -54.8 g), with no systematic patterns between the FFQ and 3DDR. No differences were found between food categories estimated with the FFQs on both occasions, and Spearman’s correlation coefficients ranged from 0.664 to 0.712 (*p* < 0.01). Cronbach’s alpha values (α > 0.700) indicate good internal consistency, and ICCs (range 0.724–0.826; *p* < 0.01) indicate good reproducibility of the FFQ.

**Conclusion:**

The results indicate reasonable relative validity and acceptable reproducibility of the FFQ for estimating FV consumption among school-aged children.

## Introduction

Recently, epidemiological studies are more focused on measuring food groups, rather than energy and nutrient intake, to observe the relation between diet and health outcomes (1, 2). Probably the most studied food groups over time are fruit and vegetables. The reason for this may be the growing number of interventions resulting from an awareness of the health benefits of fruit and vegetable consumption (3–7). At the same time, some research studies have shown a decrease in fruit and vegetable consumption (8–10). Food frequency questionnaires (FFQs) and short food questionnaires (SFQs) are commonly used to assess fruit and vegetable consumption because they are easy to complete, do not take much time, and reduce participant burden compared with food dietary records or 24-h recalls. They are also less expensive and less time-consuming to analyze (1, 11). Current questionnaires used in various studies and interventions differ in the definition of fruit and vegetables, the preparation of fruit and vegetables (fresh, canned, frozen, cooked, etc.), the serving size, the units of serving size, and reported frequencies. Moreover, only a few of them have been tested for validity and reproducibility and are suitable for assessing fruit and vegetable consumption in school-aged children (12–14).

This study reports on the development of an FFQ measuring fruit and vegetable consumption in school-aged children (7–10 years). Previous research in Croatia has shown data on the consumption of fruits and vegetables (10), and there is a need for such a tool to assess actual fruit and vegetable consumption. The data obtained on fruit and vegetable consumption would make it possible to design and implement targeted educational interventions, create an environment that encourages higher fruit and vegetable consumption, and monitor the progress of the interventions. In addition, the need for the development of a new FFQ arose from the cultural differences and different eating habits of children in Croatia compared to Western European countries (1, 15). Although the consumption of fast food is increasing, people in Croatia and some other Central and Eastern European countries still consume home-cooked meals prepared mainly from unprocessed and minimally processed foods (16, 17). Moreover, children from 7 to 10 years spend up to 8 h in school, so schools have to provide them with breakfast, lunch, and snacks. Parents can choose the number of meals, but all students eat the same meal items (one-meal option) in their school. Most school lunches are cooked and include composite meals such as vegetable stew, soup, and pasta with vegetable and meat sauce. Since vegetables are mostly found in composite dishes in Croatian school-aged children, it is suggested to include them in the FFQ, as well as fruit and vegetables related to the geographical and cultural environment (15). The school food procurement system can influence the availability and, consequently, consumption of fruits and vegetables by children. In Zagreb primary schools, the most commonly offered fruit categories were pome fruits (mainly apples and pears) and tropical fruits (mainly bananas and pineapple compost), and the vegetable categories were onions (mainly onions and garlic) and root vegetables (mainly carrots, parsley, and celery) (18). In addition to food procurement, participation in the school scheme program can affect the availability of fruits and vegetables. Participating schools receive free of charge 100–150 g of fresh vegetables and fruit per student once a week throughout the school year. Schools can choose their favorite fruits (apples, pears, peaches, apricots, plums, cherries, figs, tangerines, grapes, strawberries, raspberries, and blackberries) and vegetables (carrots, radishes, kohlrabi, and tomatoes) from the offer (19). The novel FFQ needs to be adjusted for children (e.g., indicating portion size, relevant frequency, and detailed description of foods) so that they can complete it with the help of their parents/caregivers as they do not have the cognitive skills to identify their food consumption, while at the same time, they need to identify food consumption outside of the home (e.g., in school) (20–22).

Once development is complete, the new FFQ must be tested for validity and reproducibility. The validity of the FFQ will ensure that it provides accurate dietary information, while reproducibility will ensure that the FFQ provides the same results without error in repeated measurements (1, 23). Accordingly, the aim of this study was to evaluate the validity of a semi-quantitative FFQ for measuring fruit and vegetable consumption in school-aged children (7–10 years) by validating it using a 3DDR. In addition, the aim was to determine the reproducibility of the new FFQ.

## Materials and methods

### Study design and ethics

The study was conducted to develop a semi-quantitative FFQ which measures fruit and vegetable consumption among school-aged children and to evaluate its validity and reproducibility. The study protocols were designed and performed in accordance with the Declaration of Helsinki, and ethical approval was obtained from the Ethics Committee of the School of Medicine, University of Zagreb (380-59-10106-19-11/307). The study was a part of the “Pilot Project: School meals and fruit and vegetable intake in schools with and without a garden” within the Horizon 2020 project “Strengthening European Food Chain Sustainability by Quality and Procurement” (Strength2Food, H2020-SFS-2015-2, contract no. 678024). All permissions for the implementation of the pilot project in schools were obtained from the relevant institutions (the Ethics Committee of the Institute for Medical Research and Occupational Health: 100-21/16-8; the Croatian Ministry of Science and Education and the Education and Teacher Training Agency: 602-01/16-01/00388).

The validity of the FFQ was assessed by comparing the results of the FFQ with the results of the 3-day dietary record (3DDR) as a reference method for analysis. The FFQ and the 3DDR were distributed to each participant while they were attending second grade in primary school (school year 2019/2020). The FFQ was distributed before 3DDR in order to reduce participant burden and the possibility of increased correlation due to awareness of diet quality from completing a 3DDR. The 3DDR was used for greater statistical efficiency because it minimizes daily variation in food consumption and has the lowest correlation errors (1). For reproducibility analysis, the second administration of the FFQ to each participant was carried out while they were attending third grade in primary school (2020/2021 school year). The time interval of 1 year was chosen to avoid the possibility of remembering the response to the first FFQ and that to be sufficiently time spaced from the administration of the 3DDR. Although a longer interval (6–12 months) may contribute to lower reproducibility because of changes in dietary habits, it was used in one-third of the reproducibility studies (1, 23).

### Participants

The study was conducted in 14 primary schools from the city of Zagreb. Schools were selected according to the protocol of the *Strength2Food* project (24). Schools from different parts of the city (center and suburbs) and from more and less affluent areas were included in the study. Of a total of 1,036 s grade children (7–8 years), parents/caregivers of 681 of them gave their written informed consent to participate in the study. The first FFQ was completed by 393 children (58% of total sample), and the 3DDR was completed by 195 children (29% of total sample). The validation study was conducted on children who completed both the first FFQ and the 3DDR, which had a final sample size of 141 children (21% of total sample). A minimum of 117 children were required to have 80% power at the α = 0.05 significance level to detect a Spearman correlation coefficient of 0.3, based on similar studies validating an FFQ with a 3DDR (25). The second FFQ was completed by 166 children (24% of total sample), five of whom were not included in the analysis because they did not complete an FFQ on the first occasion. Finally, the reproducibility study was conducted on 161 children (23% of total sample) who completed the FFQs on both occasions. A minimum of 116.7 children were required to have 80% power at the α = 0.05 significance level to detect an acceptable level of reliability (intraclass correlation coefficient of 0.70, based on similar studies) between two FFQs (26). In addition, sample sizes for both analyses were in accordance with the recommendations from Willet (1).

### Food frequency questionnaire development

Within the project *Strength2Food*, we developed the semi-quantitate FFQ to assess fruit and vegetable consumption in school-aged children. To define food groups and serving size, we first analyzed annual menus from all 14 primary schools (2,379 breakfasts, 2,376 lunches, and 1,223 snacks) since most children in Croatia eat one to three school meals daily. In addition, the most frequent lunches (*n* = 140) were weighted to verify the agreement between menus and served food. In the second step, we examined available national adult consumption data to observe eating habits regarding frequencies of fruit and vegetable consumption, which may be indicative of children’s eating habits at home (6, 27). Accordingly, the FFQ consists of five questions on fruit consumption (fresh fruits, dried fruits, fruit juices, cooked fruits, and nuts) and 13 questions on vegetable consumption (vegetable stews, legume stews, cooked/baked/grilled vegetables, cooked vegetable and potato side dishes, fresh green leafy vegetables, fresh vegetables, canned vegetables, cooked/baked/fried potato, vegetable risotto, pasta with vegetable sauce, vegetable juices, and legume spreads). The questions on nuts and potato dishes are included to prevent participants from reporting them as fruit and vegetables as they are not included in the calculation of the final FFQ results. This semi-quantitative FFQ is designed to prompt participants to reflect on the frequency of food and beverages consumed in the last month. Available frequencies in the FFQ are never, 1–3 times per month, once a week, 2–4 times per week, 5–6 times per week, once a day, 2–3 times per day, and 4–6 times per day (1). The classification of fruits and vegetables into eight different categories (“fruit and fruit juices,” “fruit,” “vegetables, vegetable juices, and legumes,” “vegetables and vegetable juices,” “vegetables,” “fruit, fruit juices, vegetables, vegetable juices, and legumes,” “fruit, fruit juices, vegetables, and vegetable juices,” and “fruit and vegetables”) makes it possible to compare the results of the new FFQ with different dietary guidelines. The estimated consumption of fruit and vegetables is expressed in gram units so that they can be compared with the recommendations of the World Health Organization (WHO) (3).

The FFQs on both occasions were distributed to parents/caregivers of enrolled children as an online questionnaire. Each question on the frequency of usual food group consumption includes the description of the food group and the amount of food and drink consumed, expressed as a serving which is explained in detail as a quantitative measure and household unit. The parents/caregivers were advised to complete the questionnaire with the children to gain better insights into their frequency of fruit and vegetable consumption while the children were out of their supervision. As FFQs were online questionnaires, they were set up so that all questions had to be answered. Therefore, children who completed the FFQ had answered all the questions, and there were no missing data from the FFQs. The FFQ takes approximately 15 min to complete.

### 3-day dietary records

The parents/caregivers along with the children recorded their children’s consumption of all foods and beverages for 3 non-consecutive days (2 weekdays and 1 weekend day). Both parents/caregivers and children were instructed on how to keep the 3DDR and given written instructions and video materials. They were instructed to weigh the number of foods and beverages or to use standard household units if they were unable to weigh the food. They were asked to weigh raw foods separately in composite meals and indicate the brand of the food products. They were also required to provide information on the time, type, and place of the meal consumption and how it was prepared. When children eat a school meal, they have been instructed to record the type of school meal and the amount of each meal component that the children ate as a percent of the served portion. After data collection, all collected 3DDRs were reviewed by the research team. The review identified errors such as missing items, duplicate items, and items without measures or brand specifications. 3DDRs that needed improvement were returned to parents/caregivers along with instructions on what should be revised. In 3DDRs, all household units were converted into gram units by the research team. The percentage of foods and beverages consumed from school meals was converted to gram units using school meal recipes to which the research team had access. The amounts of foods and beverages from 3DDR of each child were analyzed using the software “Prehrana” (Infosistem, d.d). Excel spreadsheets were extracted from the software in which the data on fruit and vegetable consumption were divided into the same categories as in the FFQ and calculated as average values of 3 days to obtain results at the same level. The mean daily energy intake for each child was estimated from the 3DDR using the same software.

### Anthropometric measurements

Anthropometric measurements were carried out by a trained member of the research team during physical education and health classes while children were wearing light athletic clothing. Anthropometric measurements included measuring of body height to the nearest 0.1 cm and measuring body weight to the nearest 0.1 kg using a combined medical digital scale and stadiometer (Seca, Type 877-217, Vogel & Halke Gmbh & Co., Germany). The body mass index (kg/m^2^) of each child was calculated from height and body weight data. The WHO sex-standardized z-scores for body mass-for-age, body height-for-age, and body mass index-for-age of each child were obtained using AnthroPlus software and used to determine the nutritional status (28, 29).

### Data analysis

Statistical analyses were performed using IBM SPSS version 23(IBM SPSS Statistics for Windows, version 22.0. Armonk, NY: IBM Corp.). A significance level of *p* < 0.05 was used for all analyses. Data were non-normally distributed according to the Kolmogorov–Smirnov test. The crude data were log-transformed to improve normality; however, the data were still skewed. Therefore, non-parametric statistical methods were applied to the crude data. Fruit and vegetable consumption estimated from the 3DDRs and the two FFQs is presented as median and interquartile ranges.

For validity analysis, the Wilcoxon signed rank test was used to determine whether the FFQ1 overestimates or underestimates fruit and vegetable consumption from the 3DDR (30). Spearman’s rank correlation coefficients (-1.0 to -0.8 = strong negative correlation; -0.8 to -0.6 = good negative correlation; -0.6 to -0.3 = moderate negative correlation; -0.3 to -0.1 = weak negative correlation; -0.1 to 0.1 = no correlation; 0.1–0.3 = weak positive correlation; 0.3–0.6 = moderate positive correlation; 0.6–0.8 = good positive correlation; 0.8–1.0 = strong positive correlation) of fruit and vegetable consumption between the 3DDR and FFQ1 were calculated to determine the linear relationship between the two methods (1, 30, 31). The ratios of within-person variance to between-person variance in fruit and vegetable consumption estimated by 3DDR were used to calculate the de-attenuation of cured correlation according to the equation $r_{a\,d\,j\,u\,s\,t\,e\,d}=r_{o\,b\,s\,e\,r\,v\,e\,d}\,\sqrt{1+\frac{\lambda_{\text{x}}}{{\text{n}}_{\text{x}}}}$ from Willet (1). Fruit and vegetable consumption estimated using the FFQ and 3DDR was ranked into quartiles using a visual binning method. Cross-classification analysis was used to examine the proportion of children who were classified in the same quartile or were extremely misclassified. Agreement between the 3DDR and the FFQ1 was examined by calculating weighted Cohen’s kappa coefficients (κ_*w*_) (32). The ranges of kappa were interpreted according to the strength of agreement categories: < 0 = poor agreement; 0.00–0.20 = slight agreement; 0.21–0.40 = fair agreement; 0.41–0.60 = moderate agreement; 0.61–0.80 = substantial agreement; and 0.81–1.00 almost perfect agreement (33). The limits of agreement (LOA) and bias between the 3DDR and the FFQ1 were calculated using the Bland–Altman analysis (34). Fruit and vegetable consumption results were plotted as mean differences between the 3DDR and FFQ1 against the mean between the 3DDR and FFQ1. It was expected that 95% of the differences between the 3DDR and FFQ1 would be within the LOA (median, 2.5th percentile, 97.5th percentile) (35).

Reproducibility of the FFQ was assessed in four steps. Differences in fruit and vegetable consumption estimated by the FFQ on both occasions were tested using the Wilcoxon signed rank test (30). Spearman’s rank correlation coefficients of fruit and vegetable consumption were calculated to determine the linear relationship between FFQ1 and FFQ2 (1, 30, 31). Cronbach’s alpha (α) test was used to measure the internal consistency of fruit and vegetable consumption estimated with the FFQ on both occasions (36, 37). Acceptable α values are between 0.70 and 0.95, while α > 0.95 is desirable (38). Intraclass correlation coefficients (ICCs: < 0.5 = poor reliability; 0.50–0.75 = moderate reliability; 0.75–0.90 = good reliability; > 0.90 = excellent reliability) with their 95% confidence intervals were calculated using a two-way mixed model and absolute agreement type to estimate the similarity of fruit and vegetable consumption between FFQ1 and FFQ2 (39–41).

## Results

The general baseline data for the participating children are shown in Table 1. Validity analyses were conducted in 141 children (51.4% of boys), whereas reproducibility of the FFQ was tested in 161 children (53.4% of boys). The children in both analyses were on average 8 years old (7, 8). In both studies, most children live in suburban areas of the city and come, almost equally, from more affluent and less affluent areas.

**TABLE 1:** Baseline characteristics of samples for validity and reproducibility studies.^a^

Characteristic	Validity (*n* = 141)	Reproducibility (*n* = 161)
**Age (yr.)**	8 (7–8)	8 (7–8)
**Sex**		
Boys (%)	51.4	53.4
Girls (%)	49.6	46.6
**City location**		
Center (%)	14.2	20.5
Suburbs (%)	85.8	79.5
**Poverty rate^b^**		
Poor (%)	40.5	38.5
Average (%)	26.2	31.7
Wealthy (%)	33.3	29.8
**Energy intake (kcal)^c^**	1,701 (1,427–1,922)	1,748 (1,428–2,028)
**Anthropometric characteristics^d^**		
Body mass-for-age z-score	-0.52 (-0.10–1.29)	0.52 (-0.09–1.22)
Body height-for-age z-score	0.85 (0.25–1.28)	0.72 (0.29–1.22)
Body mass index-for-age z-score	0.14 (-0.49–0.90)	0.28 (-0.48–092)

^a^Continuous data are reported as median (and interquartile range) and categorical data as percentage. ^b^Poverty rate of the residential area was estimated by the Croatian Bureau of Statistics. ^c^Energy intake was estimated in all children in the validity study (n = 141) and in 82 children (50.9%) who also completed a 3-day dietary record in the reproducibility study. ^d^Anthropometric characteristics were measured for 130 children (92%) in the validity study and for 135 children (85%) in the reproducibility study.

**FIGURE 1:**
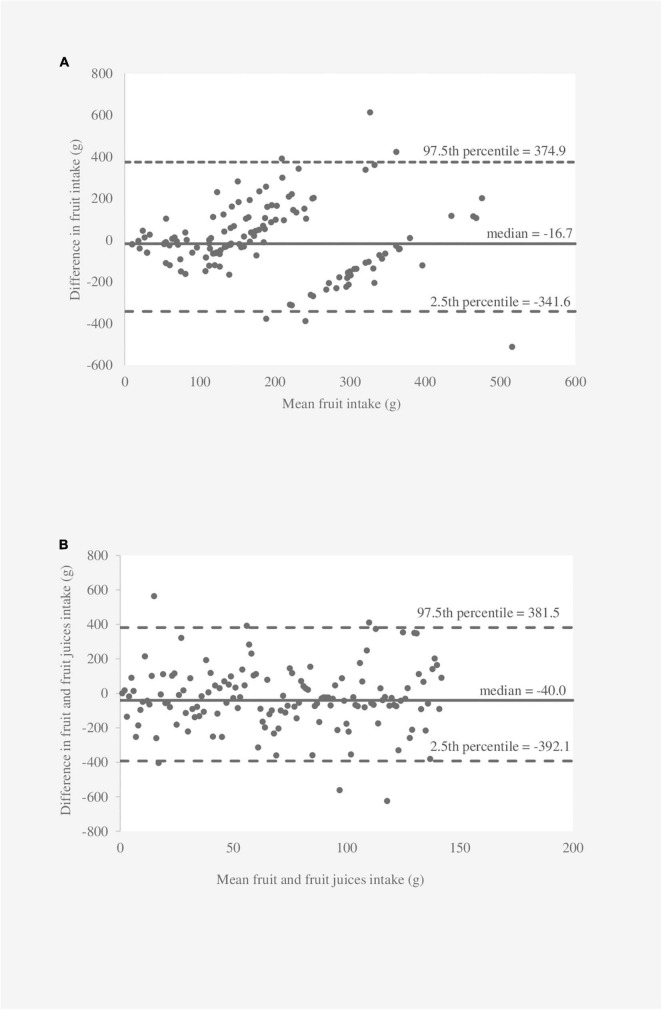
Bland–Altman plots showing agreement between the 3-day dietary records and the first food frequency questionnaire for consumption of **(A)** “fruit” and **(B)** “fruit and fruit juices” per day.

**FIGURE 2:**
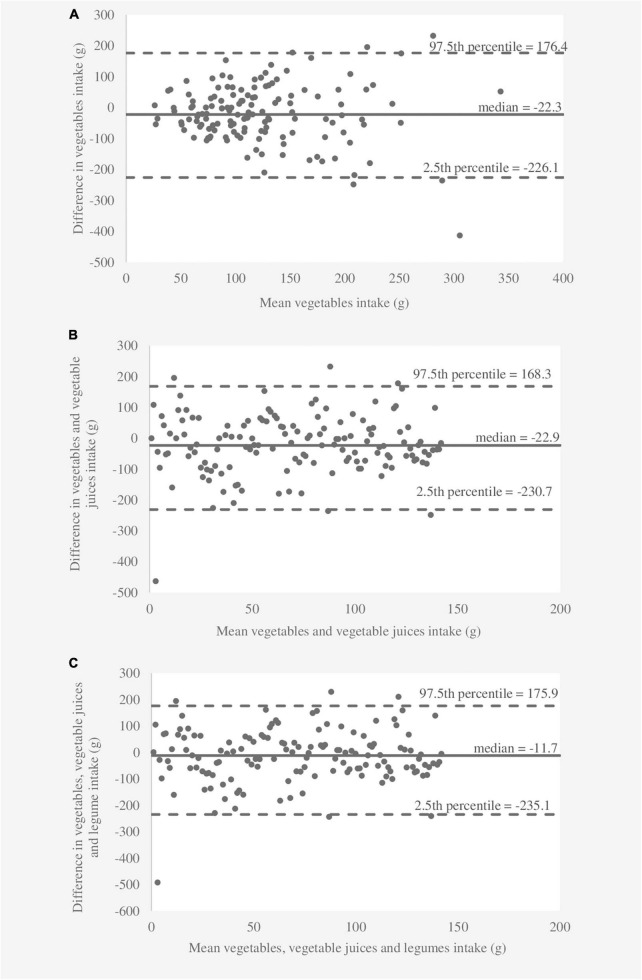
Bland–Altman plots showing agreement between the 3-day dietary records and the first food frequency questionnaire for consumption of **(A)** “vegetables,” **(B)** “vegetables and vegetable juices,” and **(C)** “vegetables, vegetable juices, and legumes” per day.

**FIGURE 3:**
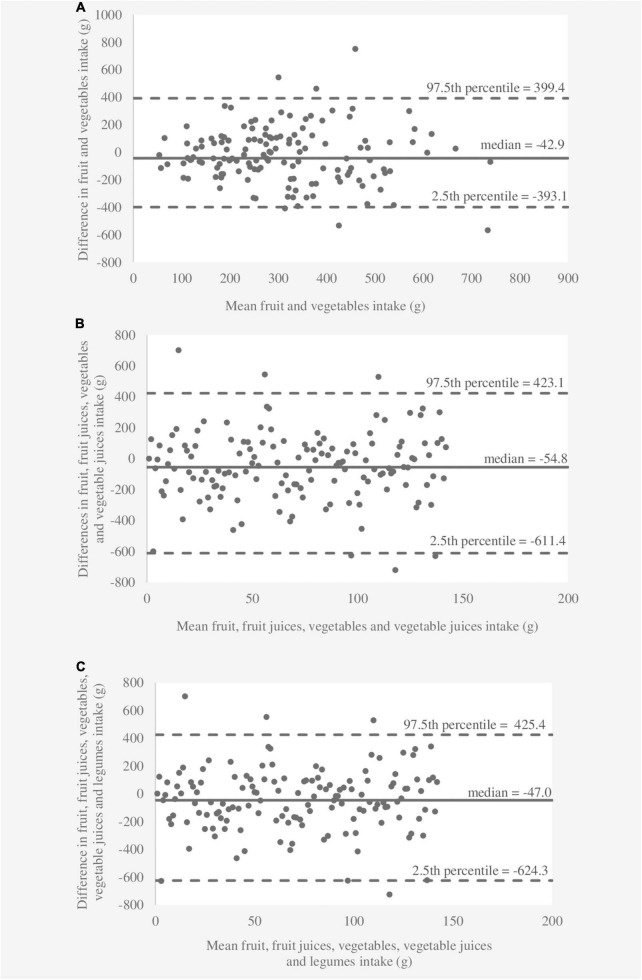
Bland–Altman plots showing agreement between the 3-day dietary records and the first food frequency questionnaire for consumption of **(A)** “fruit and vegetables,” **(B)** “fruit, fruit juices, vegetables, and vegetable juices,” and **(C)** “fruit, fruit juices, vegetables, vegetable juices, and legumes” per day.

The results of the relative validity analyses are shown in Table 2. The medians of six food categories differed significantly (*p* < 0.05) between the 3DDR and FFQ1 according to the Wilcoxon signed rank test, while the medians for consumption of “fruit” and “fruit and vegetables” were similar (*p* > 0.05). The FFQ overestimated and underestimated three food categories compared to the 3DDR. Spearman’s correlation coefficients in absolute values were significant between 0.199 and 0.362 for the results obtained from the 3DDR and FFQ1. De-attenuated correlation coefficients were slightly higher (0.217–0.384) after correcting within-person variance for between-person variance. Lower de-attenuated correlation coefficients (< 0.250) were observed for “fruit, fruit juices, vegetables, vegetable juices, and legumes” and “fruit, fruit juices, vegetables, and vegetable juices.” The average de-attenuated correlation coefficient for all food categories was 0.317. Cross-classification analysis revealed that a total of 28–41% of children were classified in the same quartile, whereas less than 10% of children were extremely misclassified for all food categories obtained from the 3DDR and FFQ1. The κ_*w*_ values showed fair agreement for all food categories. The results of the Bland–Altman analysis are shown in Figures 1–3. The differences between the 3DDR and FFQ1 were relatively small for all food categories (median between -11.7 g and -47.0 g), except for the consumption of “fruit, fruit juices, vegetables, and vegetable juices,” where the difference was -54.8 g. The Bland–Altman plots showed good agreement between the two methods, with differences well distributed around the median for all food categories across all consumption levels. In addition, 96–98% of the differences between the 3DDR and FFQ1 of all food categories were within the LOA (2.5th percentile and 97.5th percentile). No systematic patterns were observed, except for fruit consumption (where a proportional bias was observed), showing acceptable consistency between the two methods.

**TABLE 2:** Relative validity and median values for food frequency questionnaire and 3-day dietary record for the fruit and vegetable consumption in school-aged children (*N* = 141).^a^

Food groups	3-day dietary record (g/day)	Food frequency questionnaire (g/day)	Wilcoxon Signed Rank test	Spearman’s correlation coefficient	De-attenuated correlation coefficient	Classified in the same quartile	Extreme misclassified	Weighted kappa
	
	Median (interquartile range)	Median (interquartile range)	*z*	*r*	*r*	(% children)	(% children)	κ_w_
**Fruit**								
Fruit	173.3 (66.7–266.7)	152.0 (82.0–375.0)	-0.710	0.323**	0.325**	41	7	0.235*
Fruit and fruit juices	191.7 (85.7–291.7)	168.7 (120.3–385.3)	-2.197*	0.327**	0.333**	40	7	0.260*
**Vegetables**								
Vegetables	98.0 (63.0–153.0)	120.1 (78.5–164.9)	-2.269*	0.254**	0.290**	28	8	0.216*
Vegetables and vegetable juices	98.0 (63.0–153.0)	122.9 (80.2–172.6)	-2.563*	0.224**	0.245**	30	8	0.213**
Vegetables, vegetable juices and legumes	109.6 (68.5–164.7)	124.6 (83.6–177.7)	-1.703*	0.199*	0.217*	28	10	0.239*
**Fruit and vegetables**								
Fruit and vegetables	283.7 (181.7–939.3)	279.0 (188.0–469.6)	-1.363	0.354**	0.369**	31	7	0.234**
Fruit, fruit juices, vegetables and vegetable juices	321.1 (227.6–528.6)	317.8 (224.2–525.1)	-2.742*	0.348**	0.370**	33	7	0.234**
Fruit, fruit juices, vegetables, vegetables juices and legumes	316.0 (218.3–431.4)	304.0 (193.9–429.1)	-2.370*	0.362**	0.384**	33	7	0.245**

^a^Continuous data are reported as median (and interquartile range) and categorical data as number or percentage. *Difference is significant at p < 0.05. **Difference is significant at p < 0.01.

The results of the reproducibility evaluation are shown in Table 3. The Wilcoxon signed rank test revealed no evidence (*p* > 0.05) of a difference between the medians for all food categories at FFQ1 and FFQ2. The relationship between FFQ1 and FFQ2 was significant for all food categories (*p* < 0.01), and Spearman’s correlation coefficients ranged from 0.664 to 0.712. Cronbach’s alpha values were above 0.70, indicating good reliability between FFQ1 and FFQ2 for all food categories. According to the ICC (range 0.724–0.745; *p* < 0.01), moderate reliability is found for consumption of “fruit and fruit juices,” “vegetables, vegetable juices, and legumes,” “vegetables and vegetable juices,” and “vegetables,” while good reliability (ICC range 0.756–0.826; *p* < 0.01) is observed for the consumption of “fruit,” “fruit, fruit juices, vegetables, vegetable juices, and legumes,” “fruit, fruit juices, vegetables, and vegetable juices,” and “fruit and vegetables.”

**TABLE 3:** Reproducibility and median values for food frequency questionnaires for the fruit and vegetable consumption in school-aged children (*N* = 161).^a^

Food groups	Food frequency questionnaire 1 (g/day)	Food frequency questionnaire 2 (g/day)	Wilcoxon signed rank test	Spearman’s correlation coefficient	Cronbach’s alpha	Intraclass correlation coefficient*^b^*	
	
	Median (interquartile range)	Median (interquartile range)	z	r	α	ICC	95% CI
**Fruit**							
Fruit	152.0 (110.0–375.0)	152.0 (110–330.0)	-0.746	0.664**	0.755	0.756	0.665–0.820
Fruit and fruit juices	200.0 (126.7–391.7)	180.7 (118.3–3750.0)	-1.422	0.668**	0.746	0.745	0.653–0.814
**Vegetables**							
Vegetables	126.9 (91.1–187.7)	130.6 (85.2–172.9)	-1.767	0.677**	0.738	0.735	0.624–0.808
Vegetables and vegetable juices	137.3 (89.2–189.8)	132.0 (86.8–182.7)	-2.252	0.676**	0.730	0.728	0.632–0.802
Vegetables, vegetable juices and legumes	137.5 (94.5–191.11)	136.3 (88.4–185.1)	-1.383	0.677**	0.725	0.724	0.625–0.799
**Fruit and vegetables**							
Fruit and vegetables	305.1 (214.2–494.9)	296.6 (204.6–447.6)	-1.182	0.677**	0.812	0.812	0.744–0.862
Fruit, fruit juices, vegetables and vegetable juices	356.2 (235.3–547.5)	348.7 (242.0–487.0)	-2.195	0.712**	0.827	0.825	0.760–0.872
Fruit, fruit juices, vegetables, vegetable juices and legumes	355.2 (240.1–548.1)	353.3 (245.7–490.7)	-2.004	0.712**	0.829	0.826	0.763–0.873

^a^Continuous data are reported as median (and interquartile range). ^b^ICCs were calculated using a two-way mixed model, type: absolute agreement. **Difference is significant at p < 0.01.

## Discussion

To the authors’ knowledge, this is the first national specific semi-quantitative FFQ estimating fruit and vegetable consumption among school-aged (7–10 years) children in Croatia. According to the available literature, this new FFQ is one of the few tools for estimating fruit and vegetable consumption in children that have been tested for both validity and reproducibility (42–48), and not only for validity (49–56), as shown in this study. The new FFQ estimates fruit and vegetable consumption through fresh, cooked, canned, and dried fruits and vegetables, fruit and vegetable juices, and also composite dishes because it has been observed that vegetables from composite dishes can contribute to total daily vegetable consumption in children (15, 53, 57). In total, the FFQ can be used to estimate the consumption of eight food categories. The five food categories in the FFQ results are defined according to the WHO definition of fruit and vegetables (15). However, legumes were added to three food categories to allow comparison with other fruit and vegetable FFQs (42, 43, 49, 50).

The validity of the FFQ was tested using the 3DDR as a reference method. The Wilcoxon signed rank test revealed significant differences between the 3DDR and the FFQ for six food categories, while the consumption of “fruit” and “fruit and vegetables” was equal. The FFQ overestimated the consumption of “vegetables, vegetable juices, and legumes,” “vegetables and vegetable juices,” and “vegetables” and underestimated the consumption of “fruit and fruit juices,” “fruit, fruit juices, vegetables, vegetable juices, and legumes,” and “fruit and fruit juices” compared to the 3DDR. These systematic differences in fruit and vegetable consumption between the reference method (24-h recall or DDR) and the questionnaire tested (FFQ/SFQ/questionnaires) were observed in most studies, with the FFQ most frequently overestimating fruit and vegetable consumption (42, 45, 47, 48, 52–55). It has been suggested that the pre-defined serving size of the foods and beverages consumed may be the cause of the differences between the methods (42); however, the pre-defined serving size helps children and parents/caregivers to better report fruit and vegetable consumption using the FFQ (20–22). Despite the differences in medians of food categories, the de-attenuated correlation coefficients in the present study suggest a weak to moderate relationship between the FFQ and 3DDR. Consistent with these results, the observed de-attenuated correlations in other validation studies ranged from 0.09 to 0.40 (42, 47, 54, 56), although some tested questionnaires for estimating fruit and vegetable consumption showed a better relationship (>0.40) with the reference methods (53, 55). Several studies have reported crude correlation coefficients, finding a moderate (45, 51, 52) and a low (48) relationship between questionnaires and reference methods. Lower correlation coefficients between two methods were generally observed in children than in adults, especially for fruit and vegetable consumption (1). This could be due to children’s inability to report their consumption and limited parental reporting of fruit and vegetable consumption outside of the home (21). According to the cross-classification analysis, approximately one-third of the participants in the present study were classified in the same quartile of the eight food categories of the FFQ on fruit and vegetable consumption, and < 10% were extremely misclassified. This suggests that the FFQ has limited ability to discriminate fruit and vegetable consumption between quartiles. Findings from cross-classification analysis of this FFQ are similar to others (42, 54). However, in one study, the FFQ was found to be better at discriminating fruit consumption, but not vegetable consumption (51). In this validation study, a fair agreement between the FFQ and 3-DDR was estimated using κ_*w*_ values, which is consistent with the study of conducted by Bel-Serrat et al. (42), whereas a slight agreement between the FFQ and reference methods was observed in two other studies (51, 54). Only one of the available studies found poor validity between scores for fruit and vegetable consumption estimated using a short food survey and 24-h recall as the reference method, but agreement analysis was observed with ICCs (45). The lower agreement between methods could be due to the daily variability in children’s eating habits (1, 21). Also, the lower agreement may occur due to the pre-definition of serving size (42). The FFQ, like other methods for assessing food and beverage consumption in nutritional epidemiological studies, has some limitations, and bias may occur (1). Therefore, the Bland–Altman plot is a useful analysis to show the extent of bias and whether it persists across the consumption range. Nevertheless, the use of Bland–Altman analysis in validation studies of food group consumption conducted on school-aged children is low (45, 53, 55). In the present study, the Bland–Altman analysis revealed relatively small differences in all consumption levels between the 3DDR and FFQ without systematic patterns. The results from other studies were inconclusive. Lim et al. (53) observed for both DILQ and FVQ wide limit of agreements, great variability, and systematic differences in mean consumption compared with the reference method. Hunsberger et al. (55) estimated a good agreement between the Bloc FFQ and 24-h recalls for fruit and vegetable consumption, although there was no systematic difference between the two methods for vegetable consumption, whereas for fruit consumption, a great variability was found with respect to the lower average consumption. Furthermore, Hendrie et al. (45) found that bias in vegetable consumption scores was high but constant across the range of scores, while bias in fruit consumption scores was low but decreased as the indicator score increased. The differences between the results of the Bland–Altman analysis in studies may be due to the age of the participants and their ability to complete the FFQ. In addition, it may depend on whether the children completed the FFQs and reference methods (24-h recalls or DDR) alone or with their parents/caregivers, or whether the parents/caregivers completed the questionnaires and reference methods, instead of the children. Furthermore, fruit and vegetable consumption is reported in different units (cups, scores, and grams).

In the present study, the reproducibility of the new FFQ for estimating fruit and vegetable consumption was tested on two occasions over a 1-year period. The median results were not significantly different, and a strong positive relationship was observed between the two FFQ administrations. In other reproducibility studies, the same fruit and vegetable consumption was found between two FFQ administrations using the paired *t*-test or the Wilcoxon signed rank test (43, 45, 46). Metcalf et al. (44) found no differences in fruit consumption, but higher vegetable consumption was estimated for the first administration of the FFQ than for the second administration. In addition, Domel et al. (48) estimated higher consumption of “fruit,” “vegetable soups,” “vegetables, vegetable casseroles, and vegetable salads,” “fruit and fruit and vegetable juices,” “vegetable soups, vegetables, vegetable casseroles, vegetable salads, and legumes,” and “total fruit and vegetables” at the first FFQ administration than for the second administration 1 month after. In previous studies, estimated correlation coefficients (fruit: 0.36–0.82; vegetables: 0.44–0.78) differed by type of questionnaire results (43, 44, 48). Only one study estimated total fruit and vegetable consumption, and the correlation coefficient > 0.50 was observed between two FFQ administrations (48). Other than the different types of results, the differences may be due to the different definitions of fruits and vegetables. For example, Lanfer et al. (43) included vegetables and legumes, but not fried potatoes for cooked vegetables; Metcalf et al. (44) included tubers, fried potatoes, and vegetables for vegetables (without defining the preparation method—cooked or fresh); and Domel et al. (48) included fresh and cooked fruits and vegetables and vegetables from composite dishes. Furthermore, both the differences in median consumption and the strength of the relationship between the two administrations in the reproducibility test could be influenced by the time interval between the two administrations (43). The novel FFQ tested in this study showed good reproducibility, with Cronbach’s alpha values above 0.70 for all eight food categories and ICCs of 0.724–0.745 for “fruit and fruit juices,” “vegetables, vegetable juices, and legumes,” “vegetables and vegetable juices,” and “vegetables,” and > 0.750 for “fruit,” “fruit, fruit juices, vegetables, vegetable juices, and legumes,” “fruit, fruit juices, vegetables, and vegetable juices,” and “fruit and vegetables.” Metcalf et al. (44) estimated desirable α-values (α > 0.80) for both fruit and vegetable consumption, indicating good reproducibility of the FFQ. Moderate reproducibility was observed for the ICCs for fruit and vegetable consumption (0.65 and 0.73, respectively) in only one study (45), as indicated by the available literature. The other studies that presented the results of the reproducibility tests observed fair to moderate reproducibility for fruit consumption (47) and moderate reproducibility for vegetable consumption (44, 47) using Cohen’s kappa test.

The differences between the results of this study and those of other studies in which validity and reproducibility were observed could be due to the different methodological approaches, especially in the selection of the reference method (DDR or 24-h recalls; time period between the administration of questionnaires; number of days) and the definition of the input data, such as the list of fruits and vegetables, the inclusion of composite dishes, the definition of portion size, and the unit of estimation. Differences could also arise due to the age-group of the children and the way in which the data were collected—with or without the help of parents/caregivers. Differences in data management and the use of statistical analyses may also contribute to differences between studies. Notwithstanding the differences noted between studies, the results may highlight some classic outcome areas and be helpful in drawing conclusions about the validation and reproducibility of the new FFQ.

The sample size for both validity (*n* = 141) and reproducibility (*n* = 161) analyses was adequate and can ensure sufficient statistical power (1). At baseline, the children were 8 years old, so they were 9 years old at the second FFQ administration. This age range is a critical period when children become more aware of their diet but have limited ability to determine their food consumption (20, 21, 58). On the other hand, parents/caregivers may misjudge children’s food consumption outside of the home when completing FFQs and 3DDR alone (21, 44). To reduce this bias, the FFQs and 3DDR were administered to the children’s parents/caregivers and emphasized that the parents/caregivers completed them jointly with their children. In addition, both the children and their parents/caregivers were instructed to complete the 3DDR and FFQs. Although both the FFQ and 3DDR are subjective methods in which errors can occur, we did not perform additional validation with blood plasma biomarkers because known biomarkers that correlate with fruit and vegetable intake in adults showed contradictory results in children (59). It has previously been suggested that the sex of children may influence the reproducibility and validity of the FFQs (43, 44), but in this study, boys and girls were almost equally represented. In the present study, children completed the 3DDR and FFQs with the help of their parents/caregivers, which could reduce potential bias due to children’s gender (43). Children’s diet is subject to day-to-day variations, which may contribute to the lower agreement between the FFQ and reference method (1, 20, 21). To minimize this bias, the validity of the FFQ was tested against the DDR completed for 3 non-consecutive days, of which two were weekdays to capture the effects of school nutrition and one was the weekend when parents/caregivers have better insights into children’s diets. A major strength of this study is that the FFQ measures fruit and vegetable consumption in terms of composite meals, not just fresh, canned, dried, or cooked fruits and vegetables. This provides better insights into fruit and vegetable consumption, especially because children of this age eat composite dishes at school and at home, which can account for up to 50% of daily vegetable intake (48, 53, 57). The new FFQ is designed not to depend on seasonal variations in fruits and vegetables that may occur when observed with checklists for specific fruit and vegetables. However, the ability to more accurately track changes in fruit and vegetable consumption between seasons has yet to be tested. Since it does not depend on the type of fruits and vegetables, its use should more accurately reflect changes in fruit and vegetable consumption between seasons. However, this needs to be explored in the future. Also, one of the strengths of the FFQ is that its results are presented as consumption of eight food categories that can be compared with both WHO recommendations and those that include legumes in vegetable consumption.

In conclusion, the results show moderate agreement between the FFQ and the 3DDR, with constant and relatively low bias at all levels of fruit and vegetable consumption and good reproducibility after 1 year. These results are consistent with similar validity and reproducibility studies. In addition, results suggest that the newly developed FFQ could be an accurate tool for estimating absolute fruit and vegetable intake.

## Data availability statement

The raw data supporting the conclusions of this article will be made available by the authors, without undue reservation.

## Ethics statement

The studies involving human participants were reviewed and approved by the Ethics Committee of the School of Medicine, University of Zagreb. Written informed consent to participate in this study was provided by the participants’ legal guardian/next of kin.

## Author contributions

AI: conduction of the study and analysis and interpretation of the data. AI, IR, and MB: drafting of the manuscript. All authors critically analyzed and approved the final manuscript, read and agreed to the published version of the manuscript, and contributed to the study’s conception and design.
